# Appendix R: Guidelines for Verifying and Documenting the Relationships Between Microbial Cultures

**DOI:** 10.1093/jaoacint/qsaa046

**Published:** 2020-06-04

## 1 Background

Microbial cultures are dynamic systems that can accumulate inheritable changes when propagated and/or stored in laboratory environments. These changes often affect key virulence traits that are targeted during the development and testing of medical countermeasures and pathogen detection assays. For example, laboratory-propagated *Francisella tularensis* and *Coxiella burnetii* tend to lose distinctive surface antigens that protect them from the host immune response (1–3). When cultured at 37°C, *Yesinia pestis* frequently jettisons the pCD plasmid, which encodes a number of key virulence genes associated with the bacteria’s type II secretion system (4, 5). In yet another example, laboratory-acclimated *Bacillus anthracis* is less likely to sporulate (6).

These laboratory-acquired mutations have the demonstrated potential to generate conflicting results in laboratories that are working nominally with the same strain. For example, investigators at the United States Army Medical Research Institute of Infectious Diseases (USAMRIID) found as much as a 16-fold difference in virulence of internally and externally sourced *F. tularensis* Schu S4 cultures (David Waag, personal communications) that appears to be attributed to a laboratory-acquired frame shift deletion in the known virulence determinant, FTT_0615C (7). Similarly, Molins et al. (8) noted that their version of *F. tularensis* Schu S4 exhibited decreased virulence compared to other Type A isolates.

Consequently, the research community would benefit from a consensus standard for tracking provenance of microbial stocks used in different applications. This need is especially critical for microbiologists involved in developing various health care applications, such as diagnostics, vaccines and therapeutics. Microbial reference materials used in these applications are obtained from different sources and are often not qualified/certified to the same set of standards, making it difficult for results to be confidently compared.

One way to limit and monitor the genetic drift in laboratory handled strains, in addition to minimizing the passage and handling of properly stored lots, is by encouraging researchers and culture producers to carefully document the histories of bacterial cultures and routinely screen them for divergent genotypic or phenotypic signatures. This guideline establishes a framework for investigators to use in documenting the relationships among microbial cultures used in scientific studies.

## 2 Objectives

These guidelines establish the roles and responsibilities for sponsors, performers, and culture producers with respect to the verification of relatedness among test and index cultures used in an extensible study. While not broadly enforceable, the guidelines are intended to create a framework and a set of expectations for properly qualifying and documenting the provenance of microbial cultures used in scientific studies.

## 3 Concepts and Definitions

- An *extensible study* is a research program whose results and conclusions are expected to apply equally to test and index cultures.
- In an extensible study, the *test culture* is the microbial culture derived from the index culture that is being evaluated. The *index culture* is the reference culture to which the assay results are to be applied.
- Both the test and index cultures must be *traceable cultures*, meaning that each has a unique identifier (e.g., lot/batch/subculture, etc., as appropriate) and well-documented propagation history.
- Extensible studies are generally supported by:
  1. *Sponsors*.—Establish the experimental objectives
  2. *Culture producers.—*Manufacture and characterize the study’s traceable cultures
  3. *Performers.—*Conduct the study
    These roles can be filled by the same or different organizations. However, each role has specific responsibilities with respect to the culture verification process.
- *Culture verification* is the process by which the species in a test culture is shown to be sufficiently related to that in an index culture to allow the meaningful extension of experimental results from one culture to the other. The relationship between the test and index cultures should be established via propagation history and orthogonal testing. It may also be desirable to use application-oriented testing to ensure study-specific similarities between the cultures.
- *Propagation history* describes a test culture’s step-by-step derivation from the index culture via a series of propagation events. These data are an essential part of the culture verification process because a culture’s propagation history is impossible to recover through empirical means. Furthermore, production and handling details provide important clues to the health and disposition of the culture that may not be evident through empirical observations, including potential changes in the genetic makeup.
- *Orthogonal testing* is the use of functionally independent assays to verify the genotypic and phenotypic relatedness of test and index cultures. Orthogonal testing is important for identifying genetic and physical changes that might have resulted from laboratory handling and could impact the validity of an extensible study.
- *Application-oriented testing* is designed to assess the relationship between the test and index cultures with respect to the specific genotypic or phenotypic phenomena being evaluated in the extensible study. For example, if the study relates to microbial virulence, some effort should be made to show that the virulence of the test strain resembles that of the index strain.
- *Culture verification statements* provide a convenient mechanism for documenting the relatedness of microbial cultures used in extensible studies.

## 4 Roles and Responsibilities

An extensible study’s sponsor, culture producer, and performer have distinct roles and responsibilities with respect to the culture verification process. Each of these roles and their associated responsibilities are described below and can be filled by the same or different organizations.

### 4.1 Study Sponsor

The sponsor is the entity that defines the study’s objectives. The sponsor’s principal responsibilities with respect to culture verification are to approve the index cultures to which each of the test cultures must relate and to determine the acceptable level of relatedness. These responsibilities are generally accomplished by specifying the culture producer and lot number (or equivalent designator) of each of the study’s index cultures and ruling on the adequacy of performer-supplied culture verification statements.

When selecting index cultures and approving culture verification statements, the sponsor should be aware of how their decision will impact project costs. Limited availability of singled-sourced cultures or capable culture providers can drive verification costs upwards. Furthermore, the paucity of producer-supplied provenance records and test data could make it costly, if not impossible, for the performer to adequately demonstrate the relationship between the test and index cultures.

### 4.2 Culture Producer

Under these guidelines, it is the responsibility of the culture producers, who manufacture the test and index cultures, to provide the performer with any nonproprietary information that can help the performer demonstrate the relatedness of the test and index cultures. It is understood that the culture producer may not have or be able to release all of the information that the performer needs to complete a culture verification statement. However, the lack of supporting data may drive the sponsor or performer to select different sources for their index and test cultures. Internal proprietary information and HIPAA-related materials are examples of content that may not be sharable with the performer.

### 4.3 Study Performer

Organization(s) that are tasked with conducting the scientific study fill the role of performer. It is the performer’s responsibility to demonstrate to the sponsor’s satisfaction that they are using test cultures that are sufficiently related (as determined by the sponsor) to the index cultures to support meaningful comparisons and conclusions within the scope of the extensible study. The performer meets this obligation by providing the sponsor with a culture verification statement for each of their test cultures. The verification statement summarizes and references enough data to convincingly demonstrate the provenance and empirical equivalence of the test and index cultures.

Most, if not all, of the information contained in the culture verification statement should be available from the culture producer, who likely generates provenance and test data as part of their production and quality control processes. However, in some cases the performer may be required to complete additional orthogonal or application-based testing on the test culture, as dictated by the specific study.

## 5 Implementation

The performer is expected to demonstrate, to the sponsor’s satisfaction, that their test cultures are sufficiently related to the study’s index cultures by documenting passage/subculture history, orthogonal test results, and application-specific assay outcomes in a culture verification statement. Typically, the performer will not have independent access to the records or resources necessary to fully demonstrate culture relatedness at the level recommended by this guide. Rather, they will often rely on information available from the culture producer to verify the relatedness of the test and index cultures. The culture producer will generally transmit this information to the performer via certificates of analysis, product information sheets, and direct communications.

### 5.1 Culture Verification Statement

As illustrated in *Appendix A*, a well-constructed culture verification statement should relate the test culture to the index culture via the culture’s propagation history, orthogonal test results, and optionally application-oriented test results.

#### 5.1.1 Propagation History

The culture verification statement should contain a propagation history, which describes the production and handling of the test and index cultures as well as any cultures that constitute the intervening lineage (i.e., intermediate culture). Each of these cultures should be linked to its predecessor via a documented production method. The performer should specifically identify the starting cultures, culture producer, lot number, production date, and production method of each culture in the passage history. With respect to the production method, the performer should describe the materials and methods used to derive the referenced culture from its predecessor in the lineage declaration. If the current culture is the first in the chain, the production method should clearly describe the clinical, environmental, or laboratory origin of the culture and the method used to propagate the traceable culture from that origin.

#### 5.1.2 Orthogonal Test Results

It is also important that the culture verification statement describe how available orthogonal test results address the relatedness of the test and index cultures. Orthogonal testing relies on multiple analytic techniques to compare one culture to another. Cultures can be compared with respect to morphology; genotypic and phenotypic properties; metabolic, immunological, and molecular features; molecular functions; and virulence. However, the quality of microbial verification and confidence associated with it ultimately depends on the number, type, and diversity of applied assays. Culture producers should strive for more comprehensive approaches to orthogonal testing. Minimally, orthogonal testing should include a mix of genotypic and phenotypic assays. Table 1 provides examples of various tests and the largely orthogonal categories to which they apply.

**Table 1.** Examples of orthogonal assays

Category	Example assays
Morphology	Colony plating, Gram stain
Genotypic properties	Next-generation sequencing, RFLP^a^, MLVA^b^, MLST^c^
Molecular properties	Fatty acid-based microbial identification, mass spectrometry
Metabolism	Biochemical arrays
Immunological assay response	ELISA^d^, bead-based multiplex assays, DFA^e^, IFA^f^
Molecular assay response	Real-time PCR^g^
Phenotypic traits	Phage sensitivity, motility, hemolysis
Virulence	*In vivo* studies using animal models

a RFLP = Restriction fragment length polymorphism.

b MLVA = Multilocus variable number tandem repeat analysis.

c MLST = Multilocus sequence typing.

d ELISA = Enzyme-linked immunosorbent assay.

e DFA = Direct fluorescent antibody assay.

f IFA = Indirect immunofluorescence assay.

g PCR = Polymerase chain reaction.

#### 5.1.3 Application-Oriented Test Results

While orthogonal testing is intended to uncover unexpected changes that might occur in cultured bacteria during laboratory passage and handling, application-oriented tests are used to confirm that laboratory propagation did not adversely affect properties that relate directly to the planned extensible study. Although application-oriented test results do not need to be included in the culture verification statement, incorporating such data adds scientific credibility and demonstrates that the test and index samples perform comparably on assays that relate to the specific extensible study. Table 2 provides examples of application-oriented tests that can be applied under different study objectives.

**Table 2.** Examples of application-oriented assays

Application	Example assays
Molecular assays	Target-specific sequencing, real-time PCR^a^
Immunoassays	Target-specific ELISA^b^, bead-based multiplex assays, DFA^c^, IFA^d^
Therapeutics	Virulence, antimicrobial resistance or sensitivity
Vaccines	Virulence, gene expression, host immune response

a PCR = Polymerase chain reaction.

b ELISA = Enzyme-linked immunosorbent assay.

c DFA = Direct fluorescent antibody assay.

d IFA = Indirect immunofluorescence assay.

## Participants

AOAC Stakeholder Panel on Agent Detection Assays (SPADA) Working Group for Bacterial Strain Verification:

Linda Beck (*Co-Chair*), Joint Research and Development (JRAD) supporting Joint Program Executive Office (JPEO) Joint Project Leads (JPL) Chemical, Biological, Radiological, and Nuclear Defense (CBRND) Enabling Biotechnologies (EB); Deputy Under Secretary of the Army, Test and Evaluation (DUSA TE)

Shanmuga Sozhamannan (*Co-Chair*), Logistics Management Institute supporting Defense Biological Product Assurance Office (DBPAO), JPL CBRND EB

Brian Bennett, Biological Test Division, U.S. Army Combat Capabilities Development Command (CCDC) Chemical Biological Center (CBC); Dugway Proving Ground

Cory Bernhards, CCDC CBC

Thomas Blank, National Biodefense Analysis and Countermeasures Center (NBACC)

Trevor Brown, Venesco, LLC, supporting JPEO-CBRND Joint Project Management Office for CBRN Medical

Matthew Davenport, U.S. Department of Homeland Security (DHS)

Bruce Goodwin, U.S. Department of Defense (DoD) JPEO JPL CBRND EB

Randy Hofmann, CCDC CBC

Paul Jackson, Lawrence Livermore National Laboratory (LLNL) and Los Alamos National Laboratory (LANL; Retired); Middlebury Institute of International Studies (Adjunct Professor)

Scott Jackson, National Institute of Standards and Technology (NIST)

Katalin Kiss, ATCC

Nancy Lin, NIST

Timothy Minogue, United States Army Medical Research Institute of Infectious Diseases (USAMRIID)

David Rozak, Unified Culture Collection, USAMRIID

Mark Scheckelhoff, Armed Forces Health Surveillance Branch

Sanjiv Shah, U.S. Environmental Protection Agency (EPA)

Charles Young, Johns Hopkins University Applied Physics Lab (JHU/APL)

Sharon Brunelle (Technical Consultant), AOAC INTERNATIONAL

Deborah McKenzie (Staff Liaison), AOAC INTERNATIONAL

## Review and Approval

The guidelines were reviewed by SPADA and approved on October 15, 2019.

## Disclaimer

Certain commercial equipment, instruments, or materials are identified in this paper only to specify the experimental procedure adequately. Such identification is not intended to imply recommendation or endorsement by NIST, nor is it intended to imply that the materials or equipment identified are necessarily the best available for the purpose.

## Appendix A: Example of a Culture Verification Statement

### Culture Verification Statement for *Francisella tularensis* Test Culture Lot 2425-3243

#### Summary

This culture verification statement documents the relatedness of *Francisella tularensis* test culture lot 2425-3243 (Manufacturer A; Boston, MA, USA) to the sponsor-specified index culture lot 9210-2349 (J. Smith).

#### Passage History

*F. tularensis* test culture lot 2425-3243 was derived from index lot 9210-2349 via an intermediate seed culture (lot 8434-6286). The production and handling of the test and intermediate cultures are summarized as follows.

*Lot 2425-3243*.—Derived from lot 8434-6286 by Manufacturer A on August 20, 2019, according to the following procedure. A 10 µL aliquot of thawed lot 8434-6286 was spread onto Sheep’s Blood Agar (Remel) and incubated at 35°C with 5% CO_2_ for 48 h. The bacterial growth was suspended to 1.0 McFarland unit in Tryptic Soy Broth (Remel) supplemented with 12.5% glycerol. The resulting suspension was aliquoted into 1 mL cryotubes and stored at –80°C.

*Lot 8434-6286*.—Derived from the index culture (lot 9210-2349) by Manufacturer A on July 14, 2016, using a method identical to the one described above. The manufacturer maintains 8434-6286 as seed stock for producing distributable reference materials for its customers.

*Lot 9210-2349*.—Prepared by plating a spleen homogenate from a female rabbit onto chocolate agar and incubating at 35°C with 5% CO_2_ for 48 h. A single colony pick was then propagated on fresh agar under similar conditions and resusepended in DPBS with 20% glycerol for long-term storage at –70°C. The isolate was propagated from rabbit tissue as described by J. Smith (personal communications) in September 2005 and shared with Manufacturer A on May 19, 2016.

#### Orthogonal Testing

Manufacturer A used whole genome sequencing along with Biolog and Vitek 2 GN phenotypic assays to compare *F. tularensis* test culture lot 2425-3243 with index culture lot 9210-2349. These results, which are reported in the attached Certificate of Analysis (1) and detailed on the manufacturer’s website (2), identified five single nucleotide polymorphisms in noncoding regions of the bacterial genomes and generated identical genus and species calls on the Biolog and Vitek 2 GN assays. Based on these results, we conclude that the test and index cultures are sufficiently related for the test culture to be used as a surrogate for the index culture in the sponsored study.

#### Application-Oriented Testing

The manufacturer’s Certificate of Analysis (1) reports that the test and index lots exhibit identical responses to a proprietary fluorescent tagged monoclonal antibody that similarly targets the O-antigen used in the current study’s lateral flow assay.
